# Temporal Variation and Association of Aflatoxin B_1_ Albumin-Adduct Levels with Socio-Economic and Food Consumption Factors in HIV Positive Adults

**DOI:** 10.3390/toxins7124868

**Published:** 2015-11-30

**Authors:** Pauline E. Jolly, Tomi F. Akinyemiju, Megha Jha, Inmaculada Aban, Andrea Gonzalez-Falero, Dnika Joseph

**Affiliations:** Department of Epidemiology, School of Public Health, University of Alabama at Birmingham, Ryals Building, Room 217, Birmingham, AL 35294-0022, USA; tomiakin@uab.edu (O.A.); dr.meghajha@gmail.com (M.J.); caban@uab.edu (I.A.); dreigonzalez@gmail.com (A.G.-F.); nikstt@uab.edu (D.J.)

**Keywords:** aflatoxin B_1_ levels, HIV patients, Ghana, food consumption

## Abstract

The association between aflatoxin exposure and alteration in immune responses observed in humans suggest that aflatoxin could suppress the immune system and work synergistically with HIV to increase disease severity and progression to AIDS. No longitudinal study has been conducted to assess exposure to aflatoxin (AF) among HIV positive individuals**.** We examined temporal variation in AFB_1_ albumin adducts (AF-ALB) in HIV positive Ghanaians, and assessed the association with socioeconomic and food consumption factors. We collected socioeconomic and food consumption data for 307 HIV positive antiretroviral naive adults and examined AF-ALB levels at recruitment (baseline) and at six (follow-up 1) and 12 (follow-up 2) months post-recruitment, by age, gender, socioeconomic status (SES) and food consumption patterns. Generalized linear models were used to examine the influence of socioeconomic and food consumption factors on changes in AF-ALB levels over the study period, adjusting for other covariates. AF-ALB levels (pg/mg albumin) were lower at baseline (mean AF-ALB: 14.9, SD: 15.9), higher at six months (mean AF-ALB: 23.3, SD: 26.6), and lower at 12 months (mean AF-ALB: 15.3, SD: 15.4). Participants with the lowest SES had the highest AF-ALB levels at baseline and follow up-2 compared with those with higher SES. Participants who bought less than 20% of their food and who stored maize for less than two months had lower AF-ALB levels. In the adjusted models, there was a statistically significant association between follow up time and season (dry or rainy season) on AF-ALB levels over time (*p* = 0.04). Asymptomatic HIV-positive Ghanaians had high plasma AF-ALB levels that varied according to season, socioeconomic status, and food consumption patterns. Steps need to be taken to ensure the safety and security of the food supply for the population, but in particular for the most vulnerable groups such as HIV positive people.

## 1. Introduction

The human immunodeficiency virus (HIV) suppresses the immune system leading to the development of Acquired Immune Deficiency Syndrome (AIDS). Most of the approximately 4.5 billion people living in developing countries, who are chronically exposed to aflatoxins in food [1], live in Africa and Asia where high HIV rates/numbers are also prevalent. Sub-Saharan Africa is the region of the world most heavily affected by the HIV/AIDS epidemic with nearly 1 in 20 adults living with HIV [2]; this is estimated to be about 25 million people or approximately 71% of the global total [3]. Furthermore, approximately 75% of all deaths since the beginning of the HIV/AIDS pandemic have occurred in sub-Saharan Africa. Aflatoxins are a group of extremely toxic metabolites, produced in staple food crops by the common fungi *Aspergillus flavus* and *A. parasiticus*, that have been shown to impair the immune system in animal studies [4,5,6,7,8,9,10] resulting in increased susceptibility to infections, reactivation of chronic infections [8,11,12,13,14,15,16,17,18], and decreased antibody responses to vaccination [8,18]. In human studies, we found alterations in immune parameters indicating that aflatoxin exposure might contribute to impairment of the immune system [19,20]. Chronic dietary exposure to aflatoxin has also been shown to interfere with metabolism of proteins [21], food conversion [22], growth [23,24] and a number of micronutrients that are critical to health and immune functioning [25,26,27,28]. Since both HIV and aflatoxin suppress the immune system they could work synergistically to increase HIV disease severity and result in faster progression to AIDS. Previously, we measured aflatoxin levels in HIV positive adults at one time point and found significant associations between high aflatoxin levels and alterations in immune parameters and increase in HIV viral load [20,29,30].

Although the major aflatoxins (B_1_, B_2_, G_1_ and G_2_) occur together in various foods in different proportions [31], AFB_1_ is usually the predominant and most toxic form. Therefore, we measured AFB_1_-lysine adducts (AF-ALB) formed from the binding of AFB_1_ to the amino acid lysine in serum albumin. The presence of these adducts indicates long-term exposure (two to three months or longer) to aflatoxin [32,33]. Several studies have shown high levels of the AFB_1_-albumin adducts in the blood of people exposed to the toxin [20,34]. In Ghana and other developing tropical countries, staple crops such as groundnuts, maize and other cereals are often contaminated with levels of aflatoxin that far exceed the 30 μg/kg considered tolerable in food for human consumption by the FAO/WHO/UNICEF Protein Advisory Board [1,35,36,37,38,39,40,41]. Pre-harvest aflatoxin contamination of crops is favored by high temperatures, prolonged drought, and high insect activity, while post-harvest production of the toxin is favored by hot and humid conditions. A study conducted in Benin showed that aflatoxin levels increased in crops stored for three to five months [35]. The Ashanti Region of Ghana has a bimodal rainfall pattern and two rainy/maize growing seasons. A heavy rainy season runs from April to late June and a second rainy season runs from September through November [42]. Crops grown during the rainy seasons are often stored and consumed during the dry seasons. In Gambian children AFB_1_ adduct levels were shown to be lower during the rainy season than during the dry season [43]. Another study conducted among children in Benin, a country with agro-ecological zones and rainfall patterns similar to Ghana, showed little change in AF-ALB levels between February and June but a substantial increase between June and October [44].

This study was conducted to estimate temporal variation in aflatoxin levels of HIV positive people by analyzing AF-ALB levels in blood at baseline and at six (follow-up 1) and 12 (follow-up 2) months post-recruitment, and to examine association of AF-ALB levels with socio-economic and food consumption factors. No longitudinal study has been conducted to examine variation in AF-ALB levels among HIV positive people who are chronically exposed to aflatoxin in the diet, although changes in aflatoxin levels have been associated with immune suppression, increase in viral load, and HIV disease progression [20,29,30]. We hypothesized that aflatoxin exposure would vary according to the season/time of the year depending on whether predominantly fresh or stored food was being eaten and that socioeconomic status and food consumption factors would be associated with aflatoxin levels in participants.

## 2. Results

Table 1 shows the demographic, food acquisition and consumption data for 307 study participants. The majority of study participants were between ages 30–39 years, although male participants were slightly older compared with the females (36% of males were ≥40 years compared with 22% of females, *p* = 0.0001). Most participants were currently married and Christians. Females (28%) were significantly less likely to belong to high SES groups compared with males (52%) (*p* = 0.001). The majority of the participants (62%) reported not growing any of their food, and 55% reported buying over 20% of their food. Almost 70% reported storing maize for short periods (0–2 months), and the majority of participants (67%) reported storing less than 25% of their maize. About 66% reported consuming maize at least two times a week, although a higher percentage of females reported consuming maize at least two times per week compared with males (69% *versus* 53%, *p* = 0.05). About 45% of participants reported consuming groundnuts at least two times a week.

**Table 1:** Baseline characteristics of study participants.

Variables	Overall *n* =307 N [%]	Male *n* = 67 N [%]	Female *n* = 240 N [%]
Age			
18–29	88[28.66]	7[7.94]	82[34.17]
30–39	142[46.25]	35[55.56]	106[44.17]
≥40	77[25.08]	23[36.51]	52[21.67]
missing = 0			
Marital status			
Married	198[66.22]	43[68.25]	155[64.58]
Single	47[15.72]	14[20.63]	34[14.17]
Sep/Wid/Div	54[18.16]	7[11.11]	51[21.25]
missing = 8			
Socio-economic status			
Low	93[33.1]	11[18.33]	82[37.11]
Middle	95[33.81]	18[30.0]	77[34.84]
High	93[33.1]	31[51.67]	62[28.05]
missing = 26			
Religion			
Christian	258[86.29]	51[82.46]	207[87.34]
Muslim/others	41[13.71]	11[17.74]	30[12.66]
missing = 8			
Proportion of food grown			
<20%	58[20.07]	12[19.35]	46[20.26]
≥20%	52[17.99]	14[22.58]	38[16.74]
None	179[61.94]	36[51.72]	143[63.00]
missing = 18			
Proportion of food bought			
<20%	128[45.23]	30[51.72]	98[43.56]
≥20%	155[54.77]	28[48.28]	127[56.44]
missing = 24			
Proportion of maize stored			
<25%	191[66.55]	41[68.33]	150[70.35]
25%–49%	85[29.62]	16[26.67]	69[30.40]
≥50%	11[3.83]	3[5.00]	8[3.52]
missing = 20			
Months maize stored			
0–2 months	199[69.34]	40[65.57]	159[70.35]
3–5 months	80[27.87]	17[27.87]	63[27.88]
≥6 months	8[2.79]	4[6.56]	4[1.77]
missing = 20			
Groundnut consumption			
Never	18[6.25]	6[10.00]	12[5.26]
Once or less a week	138[47.92]	30[50.00]	108[47.37]
2–3 times a week	83[28.82]	16[26.67]	67[29.39]
Everyday	49[17.01]	8[13.33]	41[17.98]
Missing = 19			
Maize consumption			
Never	4[1.39]	0[0.00]	4[1.75]
Once or less a week	94[32.64]	28[46.67]	66[28.95]
2–3 times a week	75[26.04]	11[18.33]	64[28.07]
Everyday	115[39.93]	21[35.0]	94[41.23]
missing = 19			
Drink alcohol			
No	282[93.69]	55[87.30]	227[94.58]
Yes	21[6.93]	8[12.70]	13[5.42]
missing = 4			
Drink coffee			
No	282[93.69]	58[92.06]	224[94.58]
Yes	19[6.31]	5[7.94]	14[5.88]
missing = 6			
HIV status			
AIDS [symptomatic]	3[1.06]	1[1.61]	2[0.90]
HIV infected [Non-AIDS]	280[98.94]	61[98.39]	219[99.1]
missing = 24			
HBV status			
Negative	261[87.88]	209[89.32]	51[83.61]
Positive	36[12.12]	25[10.68]	10[16.39]
missing = 10			
CD4 count			
Mean	633.303	573.62	650.17
SD	281.163	238.45 *n* = 67	290.96 *n* = 240
missing = 0			

Numbers may not always add up to 307 due to missing values.

Table 2 shows the results of unadjusted mean AF-ALB levels at baseline, follow-up time 1 and follow-up time 2 by socio-demographic and food consumption variables. The mean AF-ALB varied from 14.96 ± 15.86 pg/mg at baseline to 23.27 ± 26.63 pg/mg at follow-up time 1, and 15.32 ± 15.43 pg/mg at follow-up time 2. In unadjusted analysis, females had significantly lower aflatoxin levels compared with males (*est* = −0.30, *p* = 0.02), and participants in the middle and highest SES groups also had lower aflatoxin levels compared with the lowest SES group although the differences were not statistically significant. Participants who reported purchasing over 20% of their food (*est* = 0.25, *p* = 0.01), those who reported storing over 25% of their maize (*est* = 0.23, *p* = 0.03), and those who reported storing their maize for three to five months (*est* = 0.36, *p* = 0.001) all had significantly higher aflatoxin levels over the follow up period. In addition, participants who reported consuming groundnut (*est* = 0.27, *p* = 0.02) two to three times a week had significantly higher AF-ALB levels.

**Table 2:** Unadjusted Mean aflatoxin albumin-adduct (AF-ALB) levels (pg/mg albumin) over time by socio-economic status and food consumption practices.

Variables	Baseline [Mean AF-ALB ± SD]	FUP1 [Mean AF-ALB ± SD]	FUP2 [Mean AF-ALB ± SD]	Unadjusted Estimate (*p*-value) §
Overall n	294	169	114	-
Mean AF-ALB ± SD	14.95 ± 15.86	23.27 ± 26.63	15.32 ± 15.43	
Median AF-ALB and range	10.36; 0.2–109.9	16.7; 0.9–197.5	11.29; 0.69–76.13	
Gender				Ref −0.30 (0.02)
Males	17.59 ±13.41	21.3 ± 22.52	15.57 ± 16.12	
Females	14.28 ±16.45	23.4 ± 27.82	15.65 ± 15.36	
Socioeconomic status				Ref −0.24 (0.06) −0.18 (0.15)
Lowest	17.54 ± 19.25	21.05 ± 21.57	19.06 ± 15.27	
Middle	13.19 ± 12.09	23.21 ± 27.56	13.80 ± 14.57	
Highest	13.80 ± 15.14	19.53 ± 19.53	10.97 ± 11.16	
Proportion of food grown				Ref 0.04 (0.78) 0.02 (0.83)
None	14.07 ± 13.80	20.79 ± 17.95	15.16 ± 15.16	
<20%	16.61 ± 20.86	26.30 ± 39.76	14.92 ± 16.00	
≥20%	15.83 ± 15.99	25.07 ± 31.81	19.75 ± 17.35	
Proportion of food bought				Ref 0.25 (0.01)
<20%	12.70 ± 11.95	18.95 ± 19.40	12.53 ± 10.99	
≥20%	17.20 ± 18.65	25.60 ± 31.22	17.78 ± 16.96	
Proportion of maize stored				Ref 0.23 (0.03)
<25%	13.38 ± 15.70	22.42 ± 26.55	16.17 ± 16.24	
≥25%	17.65 ± 15.77	24.88 ± 28.62	14.22 ± 14.88	
Months maize stored				Ref 0.36 (0.00) 0.02 (0.92)
0–2 months	13.84 ± 16.04	21.74 ± 26.71	16.003 ± 16.44	
3–5 months	18.58 ± 16.04	24.27 ± 27.13	12.47 ± 6.98	
≥6 months	14.43 ± 13.11	26.40 ± 23.38	23.48 ± 17.71	
Groundnut consumption				Ref 0.27 (0.02) −0.02 (0.91)
≤1 per week	11.60 ± 9.17	12.24 ± 7.65	18.68 ± 17.16	
2–3 times a week	17.31 ± 16.19	22.27 ± 18.92	19.18 ± 20.10	
Everyday	13.42 ± 12.99	22.65 ± 18.51	16.97 ± 15.23	
Maize consumption				Ref 0.24 (0.06) 0.12 (0.31)
≤1 per week	19.25 ± 25.66	12.96 ± 11.67	6.01 ± 0	
2–3 times a week	17.42 ± 16.09	25.53 ± 34.85	13.96 ± 13.07	
Everyday	14.60 ± 15.93	19.77 ± 19.55	15.95 ± 16.64	

§ Unadjusted mean aflatoxin levels based on longitudinal models of the natural log of aflatoxin over follow-up time periods; FUP1—Follow-up time 1 (6 months post-recruitment); FUP2—Follow-up time 2 (12 months post-recruitment). Ref = referent.

**Figure 1:**
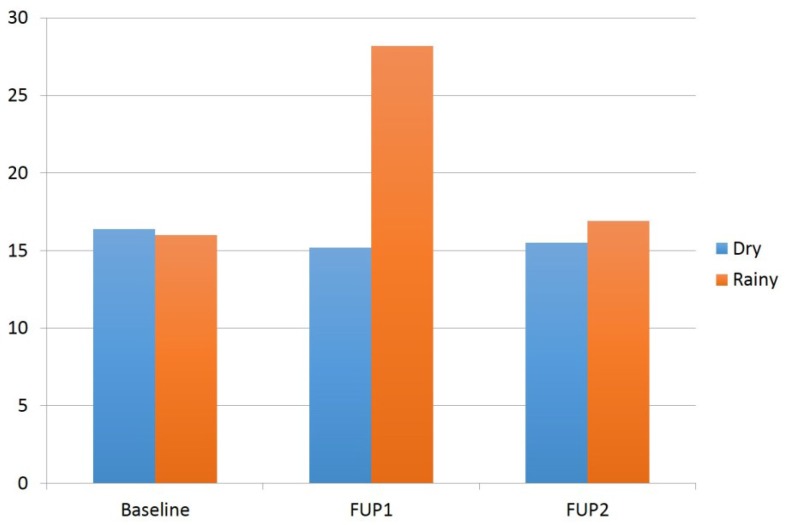
Unadjusted mean aflatoxin levels for dry and rainy seasons at baseline, follow-up 1 (FUP1) and follow-up 2 (FUP2). The increase in AF-ALB levels between baseline and follow-up 1 for the rainy seasons was statistically significant (*p* < 0.05).

In adjusted models, we observed a statistically significant association between follow-up time and season (dry or rainy season) on AF-ALB levels over time (*p* = 0.04). Table 3 presents the results of the fully adjusted interaction model including confounders such as age, gender, SES, HIV status, alcohol and smoking. Aflatoxin levels were lower in the dry *versus* rainy season during follow-up 1 (*est* = −0.46, *p* = 0.03), although this difference was not statistically significant after Bonferonni adjustment. There were also no differences in AF-ALB levels comparing dry and rainy seasons at baseline or follow-up 2. However, there was a statistically significant change in AF-ALB levels comparing follow-up 1 with baseline (*est* = 0.73, *p* ≤ 0.001) in the rainy season, but not between follow-up 2 and baseline in the rainy season. There were no significant differences over follow-up time in the dry season for follow-up 1 or follow-up 2. After accounting for the interaction between follow-up time and season, none of the food consumption or socioeconomic variables or CD4 count (Table 2 unadjusted) was statistically significant. Figure 1 shows the mean AF-ALB levels for dry and rainy seasons at baseline, follow-up 1 and follow-up 2. There was a statistically significant increase in AF-ALB levels between baseline and follow-up 1 (*p* < 0.05) restricted to the rainy seasons, but not between baseline and follow-up 2.

**Table 3:** Multivariable adjusted model of aflatoxin over follow-up time by season.

FUP	Season	Estimate	(Std. Error)	*p*-value
Baseline	Dry *vs.* Rainy	0.23	0.15	0.12
FUP 1	Dry *vs.* Rainy	−0.46	0.22	0.03
FUP 2	Dry *vs.* Rainy	0.08	0.24	0.75
FUP 1 *vs.* Baseline	Rainy	0.73	0.16	<0.001
FUP 2 *vs.* Baseline	Rainy	0.19	0.19	0.32
FUP 1 *vs.* Baseline	Dry	0.04	0.20	0.85
FUP 2 *vs.* Baseline	Dry	0.04	0.19	0.85
**FUP Season**	-	-	-	**0.04**

Model of the natural log of aflatoxin adjusted for age, gender, SES, marital status, food consumption patterns, HIV status, CD4 count, health status, alcohol and smoking; FUP—Follow-up; FUP1—Follow-up time 1 (6 months post-recruitment); FUP2—Follow-up time 2 (12 months post-recruitment).

## 3. Discussion

This study was conducted in Kumasi, the capital city of the Ashanti Region of Ghana. Thus, the majority of participants reported purchasing rather than growing most of their food. Maize and groundnuts are staple crops in Kumasi and the highest aflatoxin contamination is found in these crops [1,36,40]. However, maize is the principal crop, since groundnuts are usually eaten as a snack or included in sauces. Most of the maize eaten in this region is grown in rural areas and traded in urban and rural markets. Reports from our study participants indicate that maize purchased is quickly used; 70% reported storing less than 25% of purchased maize and storing for only 0–2 months. Two-thirds of participants reported consuming maize at least twice per week with 40% reporting daily maize consumption. Groundnut was also frequently consumed by participants, at least twice per week by 45% and everyday by 17%. The levels of the AFB_1_ biomarker found in participants from this region were higher than those reported from the Gambia [45,46] and Benin [47], but lower than levels found in areas at high-risk for liver cancer in China [32,48,49].

Our observed values of AF-ALB indicate that study participants have high levels of this AFB_1_ biomarker in their blood over time. Furthermore, mean AF-ALB levels were significantly higher at follow-up 1 compared to baseline but not between baseline and follow-up 2. This may be explained by the fact that baseline and follow-up 2 samples were collected during similar months (February–July) in 2009 and 2010, respectively, while samples for follow-up 1 were collected during August 2009 through January 2010. AF-ALB levels usually reflect two to three months of aflatoxin exposure [32,33]. The much higher AF-ALB levels in follow-up 1 over baseline indicate consumption of much higher levels of aflatoxin between recruitment and follow up 1. The three months (May–July) preceding the follow up 1 time point are part of the main growing season, at which time participants were likely eating crops harvested after the previous growing season, which were improperly dried and stored under hot, humid conditions. The stored food is likely to be the major source of aflatoxin contamination as previous studies show that storage of crops for three to five months resulted in higher aflatoxin levels [35]. 

Lower levels of AF-ALB at baseline and follow-up 2 (February–July) on the other hand, seem to reflect consumption of fresh crops harvested at the end of the September to November rainy season but also consumption of crops kept under less humid conditions during the drier months of December through March. Thus, these data seem to reflect seasonal changes at the end of the growing seasons that favor more complete drying of grains and storage under less humid conditions that keep fungal growth in check and thus less aflatoxin production. Our overall findings are similar to those of a longitudinal study conducted in Benin where the highest AF-ALB blood contamination in children was observed at the end of the rainy season in October [44]. In the Benin study, only minor changes in AF-ALB levels were observed between February and June but much higher levels were seen between June and October in three of four villages [44]. However, there is no clear explanation for the variation in the AF-ALB levels observed due to the existence of the 2 rainy (growing) seasons, the fact that maize may be stored for long periods (greater than a year), and the availability and frequency of consumption of maize, groundnuts and other types of food materials. Further studies that include collection of climatic data and more detailed identification of food sources may help to clarify the observations. 

In our unadjusted analysis, we found that participants in the middle and highest SES groups had lower AF-ALB levels compared with those in the lowest SES group, except at follow up 1 when the levels for all SES groups were not significantly different, and were higher than at either baseline or follow-up 2. Previously we found that certain socio-demographic factors, such as ethnic group, the village in which participants lived, and the number of individuals in the household, were significant predictors of high AF-ALB levels in Ghana [34]. In another study in the Kumasi area, we found that higher income, being employed, having one child, and having a flush toilet were each independently associated with a 30%–40% reduced odds of high AF-ALB levels [50]. Interestingly, participants who reported purchasing over 20% of their food, storing over 25% of their maize, and storing their maize for three to five months or longer all had significantly higher AF-ALB levels at the follow up 1 period. The purchased maize may have varying levels of contamination and storing larger quantities over a longer period (probably under hot, humid and unsanitary conditions) may result in fungal proliferation and build-up of aflatoxin levels in the food and hence higher AF-ALB. Storage of maize for 3–5 months and insect damage were found to be associated with higher aflatoxin levels in Benin [35]. 

It is encouraging to see that AF-ALB levels do not remain at the high follow-up 1 levels but dropped again to slightly higher than baseline at follow-up 2. This seems to indicate normal formation and turnover of aflatoxin albumin adducts in these asymptomatic HIV positive individuals reflecting fluctuation in aflatoxin consumption in staple foods. However, with HIV disease progression or development of liver problems (from HIV, hepatitis virus infection, antiretroviral therapy or otherwise) chronic aflatoxin exposure could contribute to faster and more severe disease progression. Chronic hepatitis B infection and ingestion of aflatoxin contaminated foods have been shown to be major risk factors for development of liver cancer [51,52]. Therefore, steps need to be taken to ensure the safety and security of the food supply for the population but in particular for the most vulnerable groups such as HIV positive people. Additionally, the people could be encouraged to examine purchased food for contamination and to sort their grains before food preparation and consumption. 

This study has certain limitations. The study sample was a convenience sample and is prone to all the limitations of convenience sampling. For instance, recruited participants are likely to be more concerned about their health and therefore healthier than non-participants inducing potential selection bias. However, we expect that this would lead to an underestimation, and not overestimation, of observed aflatoxin levels. Our inability to evaluate individual levels of maize and groundnut consumption and aflatoxin contamination at sampling time points are limitations of the study. There are also several strengths of this analysis. First, the longitudinal design enabled us to examine changes in aflatoxin levels over time, and examine seasonal patterns that would not have been possible with a cross-sectional design. Second, the use of plasma aflatoxin B_1_-lysine adducts, reflecting aflatoxin exposure in the previous two to three months, provided direct and valid measures of aflatoxin levels in the blood. Third, the relatively homogenous dietary pattern among study participants improves the generalizability of our findings to others in the population. In conclusion, HIV-positive Ghanaian adults had high plasma aflatoxin levels that varied according to season and food consumption patterns. Future studies with more detailed assessment of food harvest, storage and consumption patterns in conjunction with climatic data may help to clarify the observations. 

## 4. Experimental Section

### 4.1. Study Site, Study Participants and Data Collection

A longitudinal study among ART-naive HIV positive adults (≥18 years) was conducted in two hospitals (Kumasi South Regional and Bomso Hospitals) in Kumasi, Ghana from February 2009 to August 2010. The Kumasi South Regional Hospital (KSRH) is located between three cities (Atonsu, Agogo and Chirapatre) in the Ashanti Region and provides services to 56 communities, which consist of approximately 400,000 people. Bomso Hospital (BH) is a specialized 163 bed private hospital in Kumasi that has a comprehensive HIV care, treatment and support program. BH is in close proximity to, and works closely with, KSRH. Ethical approval for the study was obtained from the Institutional Review Board at the University of Alabama at Birmingham (UAB) and the Committee on Human Research, Publications and Ethics, School of Medical Sciences, Kwame Nkrumah University of Science and Technology (KNUST), Kumasi. After informed consent was obtained, a standardized interviewer-administered questionnaire was used to collect sociodemographic and food consumption data. The interview was conducted in private rooms at the hospitals. The medical records of patients were reviewed to obtain HIV diagnosis date and CD4+ count. Three-hundred and seven antiretroviral therapy (ART) naïve HIV positive people with CD4+ T cell counts of >300 cells/mm^3^ of blood (mean ± SD 618.10 ± 284.32; range 301–1616) were recruited and followed-up at 6 and 12 months post-recruitment. Blood samples (20cc) were collected at each time point and separated into plasma for AF-ALB (and other clinical determinations) and peripheral blood mononuclear cells for immune analyses (data to be published elsewhere). Baseline recruitment was conducted from February–July 2009, follow-up 1 from August 2009 through January 2010 and follow-up 2 from February–July 2010.

### 4.2. Determination of AFB_1_-Lysine Adducts

Plasma aflatoxin B_1_-lysine adducts, reflecting aflatoxin exposure in the previous two to three months, was measured by a modified High Performance Liquid Chromatography (HPLC)-fluorescence method [53]. Briefly, 150 μL plasma samples were digested by Pronase and loaded onto an Oasis Max cartridge from Waters Co. (Milford, MA, USA). The cartridge was sequentially washed, and eluted with 2% formic acid in methanol. The eluents were evaporated to dryness and reconstituted with 150 μL 10% methanol before HPLC analysis. HPLC analysis was carried out on an 1100 liquid chromatography system (Agilent Technologies Wilmington, DE, USA). Chromatographic separation was performed on an Agilent C18 column (5 Lm particle size, 250 × 4.6 mm). The mobile phase consisted of 20 mM ammonium phosphate monobasic (pH 7.2) and methanol in a linear gradient profile. The concentration of AFB_1_-lysine adducts was monitored at wavelengths of 405 nm (excitation) and 470 nm (emission). The peak of authentic AFB_1_-lysine adduct standard or samples was co-eluted with the retention time around 12.7 min. The detection limit of this method is 0.5 pg/mL. The results of AFB_1_-lysine adducts concentration was adjusted by serum albumin level and reported as pg/mg albumin. 

### 4.3. Statistical Analyses

Socio-demographic characteristics, food consumption patterns, and CD4 counts of participants were assessed using chi-square tests for categorical variables, fisher’s exact test for cell counts less than 5, and *t*-tests to compare group means for continuous variables. Longitudinal data analysis using mixed models in SAS was employed to determine statistically significant predictors of AF-ALB levels over time. Adjusted analysis using mixed models was conducted to adjust for socio-demographics and clinical confounders. Socio-economic status (SES) was determined using principal components analysis (PCA) on data measuring permanent household indicators such as housing type, plumbing, water and electricity. A composite SES score was obtained from PCA analysis by weighting each indicator by the coefficient of the first principal component, and each member of the household was assigned the same SES. SES was then categorized into tertiles ranging from lowest to highest SES. The distribution of AF-ALB level appeared skewed in descriptive analysis; therefore log- transformed aflatoxin values were used for all statistical models. For all analysis, *p* values ≤ 0.05 were considered as statistical significant, however, we used the Bonferonni adjustment to account for over-inflation of type 1 error due to multiple pairwise comparisons in the interaction analysis. All statistical analyses were performed with SAS 9.4 (SAS Institute Inc. Cary, NC, USA).

## References

[1] Joint FAO/WHO Expert Committee on Food Additives (JECFA) Aflatoxins: Safety Evaluation of Certain Food Additives and Contaminants.

[2] Global AIDS Overview The Global HIV/AIDS Crisis Today. PEPFAR & Global AIDS, 2013. http://www.aids.gov/federal-resources/around-the-world/global-aids-overview/.

[3] HIV Core Epidemiology Slides (2013). WHO and UNAIDS. http://www.unaids.org/en/media/unaids/contentassets/documents/epidemiology/2013/gr2013/201309_epi_core_en.pdf.

[4] Ali M.V., Mohiuddin S.M., Reddy M.V. (1994). Effect of dietary aflatoxin on cell mediated immunity and serum proteins in broiler chicken. Ind. Vet. J..

[5] Neiger R.D., Johnson T.J., Hurley D.J., Higgins K.F., Rottinghaus G.E., Stahr H. (1994). The shortterm effect of low concentrations of dietary aflatoxin and R-2 toxin on mallard ducklings. Avian Dis..

[6] Pestka J.J., Bondy G.S., Dean J.H., Luster M.I., Munson A.E., Kimber I. (1994). Mycotoxin-induced immunomodulation. Immunotoxicology and Immunopharmacology.

[7] Azzam A.H., Gabal M.A. (1998). Aflatoxin and immunity in layer hens. Avian Pathol..

[8] Gabal M.A., Azzam A.H. (1998). Interaction of aflatoxin in the feed and immunization against selected infectious diseases in poultry. II. Effect on one-day-old layer chicks simultaneously vaccinated against Newcastle disease, infectious bronchitis and infectious bursal disease. Avian Pathol..

[9] Pier A.C., Richard J.L., Thurston J.R. (1986). Immunologic changes associated with mycotoxicoses. 13. Immunomodulation in aflotoxicosis. Diagnosis of Mycotoxicosis.

[10] Venturini M.C., Quiroga M.A., Risso M.A., di Lorenzo C., Omata Y., Venturini L., Godoy H. (1996). Mycotoxin T-2 and aflatoxin B1 as immunosuppressants in mice chronically infected with Toxoplasma gondii. J. Comp. Pathol..

[11] Cysewki S.J., Wood R.L., Pier A.C., Baetz A.L. (1978). Effects of aflatoxin on the development of acquired immunity to swine erysipelas. Am. J. Vet. Res..

[12] Joens L.A., Pier A.C., Cutlip R.C. (1981). Effects of aflatoxin consumption on the clinical course of swine dysentery. Am. J. Vet. Res..

[13] Boonchuvit B.H. (1975). Interaction of aflatoxin and paratyphoid infections in broiler chickens. Poult. Sci..

[14] Byong I.Y., Rothenbacher H. (1982). Immunopathologic effects of aflatoxin on lymphoid tissues and on the pathogenesis of Newcastle disease. J. Am. Vet. Med. Assoc..

[15] Edds G.T., Nair K.P.C., Simpson C.F. (1973). Effect of aflatoxin B1 on resistance in poultry against cecal coccidiosis and Marek’s disease. Am. J. Vet. Res..

[16] Hamilton P.B., Harris J.R. (1971). Interaction of aflatoxicosis with Candida albicans infections and other stresses in chickens. Poult. Sci..

[17] Wyatt R.D., Ruff M.D., Page R.K. (1975). Interaction of aflatoxin with Eimeria tenella infection and monensin in young broiler chickens. Avian Dis..

[18] Gabal M.A., Dimitri R.A. (1998). Humoral immunosuppressant activity of aflatoxin ingestion in rabbits measured by response to Myacobacterium bovis antigens using enzyme-linked immunosorbent assay and serum protein electrophoresis. Mycoses.

[19] Jiang Y., Jolly P.E., Ellis W.O., Wang J.-S., Phillips T.D., Williams J.H. (2005). Aflatoxin B1 albumin adduct levels and cellular immune status in Ghanaians. Int. Immunol..

[20] Jiang Y., Jolly P.E., Preko P., Wang J.S., Ellis W.O., Phillips T.D., Williams J.H. (2008). Aflatoxin-related immune dysfunction in health and in human immunodeficiency virus disease. Clin. Dev. Immunol..

[21] Roebuck B.D., Maxuitenko Y.Y., Eaton D.L., Groopman J.D. (1994). Biochemical mechanisms and biological implications of the toxicity of aflatoxins as related to aflatoxin carcinogenesis. The Toxicology of Aflatoxins: Human Health, Veterinary and Agricultural Significance.

[22] Shane S.M., Eaton D.L., Groopman J.D. (1993). Economic issues associated with aflatoxins. The Toxicology of Aflatoxins: Human Health, Veterinary, and Agricultural Significance.

[23] Marin D.E., Taranu I., Bunaciu R.P., Pascale F., Tudor D.S., Avram N., Sarca M., Cureu I., Criste R.D., Suta V. (2002). Changes in performance, blood parameters, humoral and cellular immune responses in weanling piglets exposed to low doses of aflatoxin. J. Anim. Sci..

[24] Turner P.C., Collinson A.C., Cheung B.Y., Gong Y.Y., Hall A.J., Prentice A.M., Wild C.P. (2007). Aflatoxin exposure *in utero* causes growth faltering in Gambian infants. Int. J. Epidemiol..

[25] Abdelhamid A.M., el-Shawaf I., el-Ayoty S.A., Ali M.M., Gamil T. (1990). Effect of low level of dietary afaltoxins on baladi rabbits. Arch. Tierernahr..

[26] Harvey R.B., Kubena L.F., Elissalde M.H. (1994). Influence of vitamin E on aflatoxicosis in growing swine. Am. J. Vet. Res..

[27] Williams J.H., Phillips T.D., Jolly P., Stiles J.K., Jolly C.M., Aggarwal D. (2004). Human aflatoxicosis in developing countries: A review of exposure, toxicology, potential consequences and interventions. Am. J. Clin. Nutr..

[28] Obuseh F., Jiang Y., Wang J.-S., Phillips T.D., Afriyie-Gyawu E., Williams J.H., Piyathilake C., Ellis W.O., Jolly P.E. (2010). Relationship between aflatoxin B1 albumin adducts in plasma, aflatoxin M1 in urine and vitamin A and E levels in Ghanaians. Int. J. Vitam. Nutr. Res..

[29] Jolly P.E., Shuaib F., Jiang Y., Preko P.O., Baidoo J., Stiles J.K., Wang J.-S., Phillips T.D., Williams J.H. (2011). Association of viral load and liver function with high aflatoxin levels in HIV positive Ghanaians. Food Addit. Contam. Part A.

[30] Jolly P.E., Inusah S., Lu B. (2013). Association between high aflatoxin B1 levels and high viral load in HIV positive people. World Mycotoxin J..

[31] Park J.W., Kim E.K., Shon D.H., Kim Y.B. (2002). Natural co-occurrence of aflatoxin B1, fumonsin B1, and ochratoxin A in barley and maize foods from Korea. Food Addit. Contam..

[32] Wang J.S., Qian G.S., Zarba A., He X., Zhu Y.R., Zhang B.C., Jacobson L., Gange S.J., Munoz A., Kensler T.W. (1996). Temporal patterns of aflatoxin-albumin adducts in hepatitis B surface antigen-positive and antigen-negative residents of Daxin, Qidong County, People’s Republic of China. Cancer Epidemiol. Biomarkers Prev..

[33] Wild C.P., Jiang Y.-Z., Ioni G., Chapot B., Montesano R. (1990). Evaluation of methods for quantitation of aflatoxin-albumin adducts and their application to human exposure assessment. Cancer Res..

[34] Jolly P.E., Jiang Y., Ellis W.O., Awuah R.T., Nnedu O., Wang J., Phillips T., Afiyie-Gyawu E., Person S., Jolly C.M. (2006). Determinants of aflatoxin levels in Ghanaians: Sociodemographic factors, knowledge of aflatoxin and food handling and consumption practices. Int. J. Hyg. Environ. Health.

[35] Hell K., Cardwell K.F., Setamou M., Poehling H.M. (2000). The influence of storage practices on aflatoxin contamination in maize in four agro ecological zones of Benin. J. Stored. Prod. Res..

[36] Awuah R.T., Kpodo K.A. (1996). High incidence of Aspergillus flavus and aflatoxins in stored groundnuts in Ghana and the use of microbial assay to assess the inhibitory effects of plant extracts on aflatoxin synthesis. Mycopathologia.

[37] Fouzia B., Samajpati N. (2000). Mycotoxin production on rice, pulses and oilseeds. Naturwissenschaften.

[38] Carvajal M., Arroyo G. (1997). Management of aflatoxin contaminated maize in Tamaulipas, Mexico. J. Agric. Food Chem..

[39] Freitas V.P., Brigido B.M. (1998). Occurrence of aflatoxins B1, B2, G1, and G2 in peanuts and their products marketed in the region of Campinas, Brazil in 1995 and 1996. Food Addit. Contam..

[40] Kpodo K., Thrane U., Hald B. (2000). Fusaria and fumonisins in maize from Ghana and their co-occurrence with aflatoxins. Int. J. Food Microbiol..

[41] Mintah S., Hunter R.B. (1978). The incidence of aflatoxin found in groundnuts (*Arachis hypogea* L.) purchased from markets in and around Accra, Ghana. Plant Sci..

[42] Climate of Ghana Wikipedia, 2014. http://en.wikipedia.org/wiki/Climate_of_Ghana.

[43] Allen S.J., Wild C.P., Wheeler J.G., Riley E.M., Montesano R., Bennet S., Whittle H.C., Hall A.J., Greenwood B.M. (1992). Aflotoxin exposure, malaria and hepatitis B infection in rural Gambian children. Trans. R. Soc. Trop. Med. Hyg..

[44] Gong Y., Hounsa A., Egal S., Turner P.C., Sutcliffe A.E., Ha. A.J., Cardwell K., Wild C.P. (2004). Postweaning exposure to aflatoxin results in impaired child growth: A longitudinal study in Benin, West Africa. Environ. Health Perspect..

[45] Wild C.P., Hudson G.J., Sabbioni G., Chapot B., Hall A.J., Wogan G.N., Whittle H., Montesano R., Groopman J.D. (1992). Dietary intake of aflatoxins and the level of albumin-bound aflatoxin in peripheral blood in the Gambia, West Africa. Cancer Epidemiol. Biomarkers Prev..

[46] Egal S., Hounsa A., Gong Y.Y., Turner P.C., Wild C.P., Hall A.J., Hell K., Cardwell K.F. (2005). Dietary exposure to aflatoxin from maize and groundnut in young children from Benin and Togo, West Africa. Int. J. Food Microbiol..

[47] Gong Y.Y., Egal S., Hounsa A., Turner P.C., Hall A.J., Cardwell K.F., Wild C.P. (2003). Determinants of aflatoxin exposure in young children from Benin and Togo, West Africa: The critical role of weaning. Int. J. Epidemiol..

[48] Wang J.S., Shen X., He X., Zhou Y.-R., Zhang B.-C., Wang J.-B., Qian G.-S., Kuang S.-Y., Zarba A., Egner P.A. (1999). Protective alterations in phase 1 and 2 metabolism of aflatoxin B1 by oltipraz in residents of Qidong, People’s Republic of China. J. Natl. Cancer Instit..

[49] Wang J.S., Huang T., Su J., Liang F., Wei Z., Liang Y., Luo H., Kuang S.Y., Qian G.S., Sun G. (2001). Hepatocellular carcinoma and aflatoxin exposure in Zhuqing Village, Fusui County, People’s Republic of China. Cancer Epidemiol. Biomarkers Prev..

[50] Shuaib F.M.B., Ehiri J.E., Yatich N.J., Funkhouser E., Person S.D., Wilson C., Turpin C., Agbenyega T., Williams J.H., Qian G. (2012). Socio-demographic determinants of aflatoxin B_1_-lysine adduct levels among pregnant women in Kumasi, Ghana. Ghana Med. J..

[51] Qian G.S., Ross R.K., Yu M.C., Yuan J.M., Gao Y.T., Henderson B.E., Wogan G.N., Groopman J.D. (1994). A follow-up study of urinary markers of aflatoxin exposure and liver cancer risk in Shanghai, People’s Republic of China. Cancer Epidemiol. Biomarkers Prev..

[52] Ross R.K., Yuan J.M., Yu M.C., Wogan G.N., Qian G.S., Tu J., Groopman J.D., Gao Y.T., Henderson B.E. (1992). Urinary aflatoxin biomarkers and risk of hepatocellular carcinoma. Lancet.

[53] Qian G., Tang L., Wang F., Xu G., Massey M.E., Williams J.H., Phillips T.D., Wang J.S. (2013). Physiologically based toxicokinetics of serum aflatoxin B_1_-lysine adducts in F344 rats. Toxicology.

